# Enhancing population-wide administrative data to better understand mortality risk in underserved groups

**DOI:** 10.1016/j.ssmph.2026.101933

**Published:** 2026-05-14

**Authors:** Emma Ross, Sarah McKenna, Aideen Maguire

**Affiliations:** Administrative Data Research Centre Northern Ireland (ADRC NI), Queen's University Belfast, Belfast, Northern Ireland, United Kingdom

**Keywords:** Health inequalities, Mortality, LGB+, Ethnic minorities, Underserved groups, Administrative data

## Abstract

**Background:**

Underserved groups (USGs) face significant health inequalities, but studying these populations is often limited by small sample sizes and low participation in voluntary studies. Population-wide Census data provides an important opportunity to address some of these challenges. This study examined mortality outcomes among multiple USGs in Northern Ireland using Census-linked mortality data.

**Methods:**

We conducted a population-wide cohort study using the Northern Ireland Mortality Study 2021, linking Census data to death records for all individuals aged ≥16 years (n = 1,463,459) from 21 March 2021 to 31 December 2023. USGs included ethnic minorities, migrants, those with limited English, LGB+ (lesbian, gay, bisexual, and other sexual minorities) individuals, religious minorities, and Irish Travellers. Outcomes included all-cause mortality, avoidable mortality, and deaths of despair (comprising suicide, alcohol-related deaths, and drug-related deaths), with suicide also examined as a separate outcome. Cox proportional hazards models were used to estimate associations with sequential adjustment for sociodemographic and health-related factors.

**Results:**

Of 1,463,459 cohort members, 414,540 (28.3%) belonged to at least one USG. During follow-up, 36,525 (2.5%) individuals died. In unadjusted models, ethnic minorities, migrants, those with limited English, and religious minorities had lower all-cause mortality risk, whilst LGB + individuals had elevated risk (HR 1.24, 95% CI 1.20-1.29). After full adjustment, estimates remained below 1.00 for all groups except LGB + individuals. For LGB + individuals, all-cause mortality was attenuated towards the null, but deaths of despair remained elevated (HR 1.21, 95% CI 1.02-1.43).

**Conclusions:**

Most USGs had lower mortality risks than their reference populations. However, LGB + individuals had elevated risk of deaths of despair. These findings demonstrate the value of population-wide data for understanding heterogeneity in mortality across USGs.

## Highlights

• Uses population-wide Census-linked mortality data to study multiple underserved groups.
• Finds lower all-cause and avoidable mortality in most minority populations.
• Identifies persistently elevated deaths of despair risk among LGB + individuals.
• Associations varied after adjustment for social and health-related factors.
• Demonstrates how aggregated categories can mask subgroup vulnerabilities.

## 1 Introduction

Health inequalities in relation to minority and underserved groups (USGs) represent a significant public health concern, with mounting evidence suggesting that these groups may face disproportionate risks of adverse health outcomes, including elevated mortality rates (Marmot et al., 2020). Within this paper, USGs include individuals who identify as LGB+ (lesbian, gay, bisexual, and other sexual minorities), ethnic minorities and Irish Travellers (an Indigenous ethnic minority group in Ireland), religious minorities, migrants, and individuals with limited English proficiency - populations often targeted by public health policy because their structural disadvantage and marginalisation within health and social care systems exacerbate vulnerability (Espinoza & Derrington, 2021; Hatzenbuehler et al., 2024; Irizar et al., 2023; Quirke et al., 2022; Rechel et al., 2013; Staniforth & Such, 2019). The groups included in this study were selected based on existing literature on underserved and marginalised populations, their relevance within public health policy, and the availability of corresponding measures within the Census. These categories are not mutually exclusive, and individuals may belong to more than one underserved group.

Individuals who identify as LGBTQIA+ (lesbian, gay, bisexual, transgender, queer or questioning, intersex, asexual, and other identities) experience disproportionately high rates of mental illness, suicidal behaviours, and mortality compared to their heterosexual and cisgender peers (Marchi et al., 2022; Zachry et al., 2025). A recent systematic review and meta-analysis pooling over 80,000 LGBTIQ (lesbian, gay, bisexual, transgender, intersex, and queer or questioning) individuals and nearly 4 million controls found markedly higher odds of suicidal ideation (OR 3.76), suicide attempts (OR 4.36), and non-suicidal self-injury (NSSI) (OR 4.24) (Marchi et al., 2022). More recent evidence also indicates elevated mortality risks, including deaths of despair (Zachry et al., 2025). However, the evidence base summarised in these reviews relies largely on survey and clinic-based samples with limited population coverage, which restricts generalisability (Marchi et al., 2022; Zachry et al., 2025). In the UK, for example, the absence of routine data collection on sexual orientation undermines the reliability of findings and limits understanding of the social determinants of these outcomes (Harkins & Hoffman, 2024). In the present study, the term LGB+ is used because the Census captures sexual orientation but does not include a measure of gender identity.

Similar health disadvantages have been observed in the Irish Traveller community, where disproportionately high levels of physical ill-health, mental illness, and suicidality have been documented (All Ireland Traveller Health Study Team, 2010; Dagli & Webb, 2025; McKey et al., 2022). A recent systematic review highlighted that these disparities are largely driven by socioeconomic disadvantage, persistent discrimination, and barriers to healthcare access (Dagli & Webb, 2025). Supporting evidence shows elevated suicide mortality (All Ireland Traveller Health Study Team, 2010; McKey et al., 2022), frequent presentations for self-harm and suicidal crisis in emergency settings (Kavalidou et al., 2023), and poorer self-reported physical health (Kennedy et al., 2023). However, much of the literature is cross-sectional, based on relatively small samples, or focused on single outcomes rather than overall mortality, which limits the scope for robust causal inference and population-wide estimation (Dagli & Webb, 2025; McKey et al., 2022).

Beyond Travellers, other ethnic minority groups in the UK and Ireland also face health disadvantages. Research highlights poorer physical and mental health outcomes, with some evidence of elevated suicide risk linked to structural disadvantage and discrimination (Bignall et al., 2020; Forte et al., 2018). However, large-scale population studies from England and Wales show that suicide patterns vary across groups, with some minority ethnic groups having lower risk than White counterparts, while others, particularly those of Mixed ethnicity, show substantially higher risk (Knipe et al., 2024; ONS, 2023). Yet much of the wider evidence remains based on small-scale or community-based studies, which limits generalisability across populations (Bignall et al., 2020; Forte et al., 2018). Longstanding gaps in the availability and quality of routine ethnicity data, which were highlighted during the COVID-19 pandemic, continue to limit robust mortality analysis; agreement between administrative sources and Census self-report is especially poor for some groups, with disproportionately high missingness among minority ethnic populations (Bhala et al., 2020; ONS, 2024; Public Health England, 2020; Razieh et al., 2025).

In contrast to these patterns of disadvantage, evidence on religion and health is mixed. Some large observational studies and reviews suggest that religious involvement, particularly service attendance, is associated with lower risks of mental ill-health, suicide, and all-cause mortality (Chida et al., 2009; Koenig et al., 2023; VanderWeele, 2017). Other evidence, however, finds no consistent protective effect, with variation across cultural and clinical contexts (Lawrence, Brent, et al., 2016; Lawrence, Oquendo, & Stanley, 2016). A population-based study in Northern Ireland similarly found no overall reduction in suicide risk by religion, with patterns differing by denomination and age (O'Reilly & Rosato, 2015). This heterogeneity is consistent with Durkheim's theory of social integration, which emphasises group-specific cohesion and regulation (Durkheim, 1951). This is particularly relevant in Northern Ireland, where religious identity is embedded within a complex religiopolitical landscape that may influence health outcomes in ways that extend beyond traditional theoretical models. Nonetheless, the literature remains dominated by cross-sectional or clinically based studies, and robust longitudinal, population-wide cohort analyses are relatively scarce (Lawrence, Oquendo, & Stanley, 2016).

Conflicting evidence exists around the health and mortality risks of migrant populations. Some studies support the “healthy migrant effect,” where immigrants initially exhibit better health outcomes than native-born populations due to the selective nature of migration (Razum, 2008), while others demonstrate wide variation in outcomes by birthplace and host country (Aldridge et al., 2018; Ikram et al., 2016; Kearns et al., 2017; Koppenaal et al., 2003; McKay et al., 2003) However, many analyses rely heavily on death records and do not fully adjust for sociodemographic confounders such as deprivation, risking biased estimates (McKay et al., 2003; Wallace & Kulu, 2014). In addition, language barriers can exacerbate inequities by hindering access and compromising care quality for people with limited English proficiency (Espinoza & Derrington, 2021; Flores, 2006).

The culmination of various disadvantages across different groups can manifest particularly as “deaths of despair,” which include deaths from suicide, alcohol-related causes, and drug-related deaths. The concept was introduced by Case and Deaton (2017) to capture the societal toll of inequality, economic decline, and social fragmentation (Case & Deaton, 2017). Such deaths often reflect broader challenges faced by marginalised populations, including health inequalities, addiction, and economic hardship (Dasgupta et al., 2018; Marmot et al., 2020; Reeves & Stuckler, 2016). While the term is widely used in population health research, it remains contested and may oversimplify the heterogeneous causes and circumstances underlying these deaths (Darke et al., 2025). In the present study, the term is used pragmatically to describe a composite category of suicide, alcohol-related deaths, and drug-related deaths that are often examined together, rather than to imply that these deaths arise from a single underlying process of despair.

Despite the priority given to addressing health disparities in current policy (Marmot et al., 2020), significant gaps remain in our understanding of health inequalities among USGs, particularly in the UK and Ireland. As noted above, research often focuses on single minority populations or specific outcomes, restricting broader comparisons and limiting cross-cutting policy responses (Bignall et al., 2020; Irizar et al., 2023). Population-wide administrative data has become a valuable resource for monitoring mortality and health inequalities (Jutte et al., 2011; Mazzali & Duca, 2015) but gaps in the routine collection of key characteristics such as ethnicity, sexual orientation, and migrant status limit what can be achieved with these sources alone (Bohensky et al., 2010; Bozorgmehr et al., 2023; Harkins & Hoffman, 2024; Vega Perez et al., 2022). In this context, the national Census remains the most comprehensive population-level source, offering near-complete coverage and standardised measures of protected characteristics, and therefore provides an unparalleled opportunity to examine health outcomes among USGs.

This study utilises population-wide Census data from the 2021 Northern Ireland Mortality Study to examine mortality risks among a range of USGs. We aim to.

1. Quantify the risk of all-cause mortality, avoidable mortality, and deaths of despair among LGB + individuals, ethnic minorities, religious minorities, Irish Travellers, migrants, and those with limited English language proficiency, and among individuals belonging to any USG compared with those belonging to none. Death by suicide was also examined separately.
2. Examine how these risks vary across different USGs, accounting for sociodemographic factors, physical health status, and mental health conditions.

## 2 Methods

### 2.1 Study Design and data sources

We conducted a longitudinal, population-wide cohort study using data from the 2021 Northern Ireland Mortality Study (NIMS), which links responses from the 2021 population Census to death records. The study population comprised all individuals aged 16 years and above who were enumerated in Northern Ireland Assembly Research and Information Service (RaISe), 2025 Census. As the analysis was based on whole-population Census enumeration rather than a sample survey, population weights were not applied. The Census provided comprehensive demographic, socioeconomic, and health information, while mortality data were obtained from death registrations from the General Register Office Northern Ireland (GRONI). Additional area-level data were linked at the Super Output Area (SOA) level. SOAs are small-area geographical units used for statistical reporting in NI. Area-level deprivation was measured using the Income Deprivation Domain of the Northern Ireland Multiple Deprivation Measure (NIMDM) 2017, assigned to SOAs based on 2021 Census geography (Northern Ireland Statistics and Research Agency, 2017). This domain identifies the proportion of the population on low income at the small-area level. For this analysis, deprivation was grouped into quintiles ranging from least deprived to most deprived. Including area-level deprivation allowed the analysis to account for broader socioeconomic context that may not be fully captured by individual or household-level Census measures.

### 2.2 Identification of underserved groups (USGs)

The USGs examined in this study were defined based on existing literature on underserved populations, public health policy relevance, and the availability of corresponding Census variables. USGs were identified using responses to select 2021 Census questions and coded into six binary variables. Ethnic minority status was identified from question 12 of the Census which asked, ‘What is your ethnic group?’. Individuals who self-identified as any ethnicity other than 'White' were defined as ethnic minorities, with 'White' included as the reference group. Those who self-identified as 'Irish Traveller', a recognised Indigenous ethnic minority group in Ireland, were included as a standalone USG in addition to the ethnic minority variable. Migrant status was determined from question 7 of the Census which asked, ‘What is your country of birth?’. Individuals born outside the UK and Republic of Ireland were defined as migrants, while those born in the UK or Republic of Ireland formed the reference group. This classification reflects the unique constitutional status of NI under the Good Friday Agreement, which recognises the right of residents to identify as Irish or British, as well as the historical Common Travel Area that has facilitated free movement between these jurisdictions since 1922 (Ryan, 2001; UK Government, 1998). Question 24 of the Census asked, ‘Which of the following best describes your sexual orientation?’. LGB + individuals were defined as those who reported any sexual orientation other than 'Heterosexual/Straight', with 'Heterosexual/Straight' acting as the reference group. Religious status was determined from question 13, which asked, ‘What religion, religious denomination or body do you belong to?’ Individuals who reported their religion as anything other than Protestant, Catholic or Other Christian were coded as religious minorities, with Protestant, Catholic, and Other Christian as the reference group. For those with a non-response to this question, religious status was backfilled using the follow-up question, 'What religion, religious denomination, or body were you brought up in?'. This two-question approach is unique to the NI Census, reflecting the region's historical sectarian divisions where religious identity often correlates with political and cultural affiliations. Even when individuals no longer practice a religion, their religious background remains a significant social identifier. The backfilling approach therefore ensures more complete data coverage while respecting individuals' choices about current religious identification. Question 16 asked, ‘How well can you speak English?’. Those who reported speaking English 'not well' or 'not at all' were coded as having limited English proficiency, whilst those who reported speaking English 'well' or 'very well' acted as the reference group.

For analyses, each USG was compared to its respective reference group. Although the USG categories are not mutually exclusive, the present analysis examines the association between membership of each USG and mortality separately. Intersectional combinations of group membership were not examined in the current paper. We also created a composite variable (“any USG”) to capture individuals belonging to at least one USG, compared with those not belonging to any USG. This was intended to provide a broad descriptive comparison between those identified as belonging to any USG and the remainder of the population, while recognising that such aggregation may mask important differences between groups.

### 2.3 Outcome measures

Outcome measures included four mortality categories: all-cause mortality, avoidable mortality, deaths of despair, and death by suicide. These were identified using International Classification of Diseases, 10th Revision (ICD-10) codes from death certificates. Death by suicide was defined using ICD-10 codes X60-X84, Y87.0 (intentional self-harm) and Y10-Y34, Y87.2 (events of undetermined intent). Deaths of despair, a term coined by Case and Deaton (Case & Deaton, 2017), were defined using the following ICD-10 codes: suicide (as above), alcohol-related causes (F10, G31.2, G62.1, I42.6, K29.2, K70, K73, K74.0-K74.2, K74.6, K86.0, X45, Y15), and drug overdoses (X40-X44, X60-X64, X85, Y10-Y14). This outcome was used as an established composite measure in population health research, while recognising that the constituent causes of death are heterogeneous. Death by suicide was also examined as a separate outcome because it is defined by intentional self-harm and its determinants and implications differ from those of alcohol-related and drug-related mortality, which may include accidental as well as intentional causes. Separate analyses of alcohol-related and drug-related outcomes were not undertaken because the data are held within a Trusted Research Environment (TRE) and disaggregation of overlapping constituent categories would produce cell counts for some USGs that fall below the minimum thresholds permitted for release. For the same reason, the relative contribution of each constituent cause to the composite measure cannot be separately reported. Drug-related suicides were included within the suicide category where they met the suicide definition above, and within the composite deaths of despair category where they met the relevant ICD-10 criteria.

Avoidable mortality was defined as deaths occurring before age 75 from causes classified as either preventable or treatable according to the OECD/Eurostat list of avoidable causes of mortality (OECD-Eurostat, 2022). Preventable causes are those that may be avoided through effective public health and primary prevention, whereas treatable causes are those that may be avoided through timely and effective healthcare (OECD-Eurostat, 2022). A complete list of the ICD-10 codes used to identify avoidable deaths can be found in Supplementary Table 1.

Covariates were also derived from 2021 Census responses and included age on Census Day (divided into 20-year age bands), gender, area-level income deprivation quintile (derived from the Northern Ireland Assembly Research and Information Service (RaISe), 2025), housing tenure (owner-occupied, privately rented, socially rented), self-rated general health (very good, good, fair, bad, very bad), presence of limiting long-term illness (no, yes – limited a little, yes – limited a lot), and self-reported chronic poor mental health (yes/no).

### 2.4 Statistical analysis

We conducted Chi-squared tests of independence to explore the socio-demographic characteristics of USGs compared to their respective reference groups (individuals not belonging to the specific USG). Similar analyses were performed to compare the proportion of deaths from each mortality outcome between USGs and their reference groups, as well as a composite ‘any USG’ category compared with no USG membership.

A directed acyclic graph (DAG) summarising the analytical framework underpinning the sequential models is provided in the Supporting Materials (Supplementary Figure 1). In each analysis, USG membership was treated as the primary exposure and mortality outcomes as the endpoints. Age and gender were treated as baseline covariates. Income deprivation/housing tenure and health-related variables (general health/limiting long-term illness and poor mental health) were considered likely to lie on the pathway between USG membership and mortality outcomes and are therefore not treated as confounders in a causal sense. Their inclusion in the sequential models instead serves a descriptive purpose, enabling assessment of the extent to which observed mortality patterns are accounted for by differences in socioeconomic circumstances and health profiles between USGs and their reference populations. Where estimates are attenuated after adjustment, this suggests that the mortality difference is partly attributable to such differences; where estimates persist, this indicates a pattern not explained by the measured covariates. The sequential models were therefore used to examine how associations changed after accounting for these factors, rather than to estimate direct causal effects.

Cox proportional hazards models were used to estimate associations between USG membership and each mortality outcome. Cox models were used because the data were structured as time-to-event, allowing variation in follow-up time and censoring to be considered. Models were fitted sequentially: unadjusted; adjusted for age and gender; additionally adjusted for area-level deprivation and housing tenure; additionally adjusted for self-rated health and limiting long-term illness; and additionally adjusted for poor mental health. Survival time was calculated from Census Day (21 March 2021) to date of death, or end of follow-up on 31 December 2023, whichever came first. Individuals alive at the end of follow-up were censored on 31 December 2023. For cause-specific analyses, deaths from other causes were treated as censored. Information on emigration was not available, and therefore unobserved out-migration could not be separately censored. The proportional hazards assumption was assessed using Schoenfeld residuals, and no substantial violations were identified. All analyses were conducted using STATA 17.

### Ethical approval

The NIMS database has ethical approval from the Office for Research Ethics Committees Northern Ireland (ORECNI) (REC Reference Number 22/NI/0042). This study was approved by the Northern Ireland Longitudinal Study Research Approvals Group (RAG). All data were de-identified and handled in accordance with the Data Protection Act 2018 and the Northern Ireland Statistics and Research Agency's (NISRA's) data security protocols.

## 3 Results

### 3.1 Cohort overview

Of the 1,463,459 individuals aged 16 years and over included in our analysis, 36,525 (2.5%) died during the 33-month follow-up period (Table 1). Deaths included 17,300 (1.2%) avoidable deaths, 941 (0.06%) deaths of despair, and 245 (0.02%) deaths by suicide (Table 1). A total of 414,540 (28.3%) of the NI population identified as being a member of at least one of the six identified USGs. The largest USGs were religious minorities (262,330 [17.9%]) and LGB + individuals (136,429 [9.3%]), followed by migrants (104,042 [7.1%]) and ethnic minorities (41,123 [2.8%]). Irish Travellers (1553 [0.1%]) and those with limited English proficiency (17,181 [1.2%]) represented smaller proportions of the cohort.

**Table 1:** Descriptive overview of mortality outcomes across underserved groups (USGs) in Northern Ireland.

Underserved group (USG)	Total N (%)	All-cause mortality	Avoidable deaths	Deaths of despair	Death by suicide
Total cohort	1,463,459 (100)	36,525 (2.5)	17,300 (1.2)	941 (0.06)	245 (0.02)

Any USG	414,540 (28.3)	7260 (1.8)	4459 (1.1)	360 (0.1)	114 (0.03)
Ethnic minority	41,123 (2.8)	211 (0.5)	111 (0.3)	11 (0.03)	n/a
Migrants	104,042 (7.1)	683 (0.7)	350 (0.3)	39 (0.04)	12 (0.01)
Limited English Language	17,181 (1.2)	187 (1.1)	95 (0.6)	n/a	n/a
LGB+	136,429 (9.3)	4145 (3.0)	1971 (1.4)	120 (0.1)	28 (0.02)
Religious minorities	262,330 (17.9)	3112 (1.2)	1587 (0.6)	169 (0.06)	58 (0.02)
Irish Travellers^a^	1553 (0.1)	27 (1.7)	n/a	n/a	n/a

N: Total number of individuals in each group.

n: Number of deaths for each outcome.

%: Percentage of deaths within each group.

Percentages shown are descriptive only and are not age-adjusted.

n/a: insufficient numbers.

a Irish Travellers are captured within the ethnic minority group but are also analysed separately given their unique status as an Indigenous minority in Ireland.

Analysis of baseline characteristics revealed notable differences in the health and sociodemographic profiles of NI's USGs compared to their reference populations (Supplementary Table 2–7). Patterns of advantage and disadvantage varied considerably across groups. Ethnic minorities, migrants, and those with limited English proficiency shared several advantageous characteristics, including better self-rated health and a lower prevalence of limiting long-term illness compared to their reference populations. Poor mental health was also notably less common among ethnic minorities (6.1% vs 10.5% of White individuals), migrants (5.1% vs 10.8%), and those with limited English proficiency (4.5% vs 10.4%). These groups also showed younger age profiles, with approximately half of ethnic minorities and migrants aged 25-44 years. LGB + individuals and religious minorities presented more complex patterns. LGB + individuals reported a higher prevalence of poor mental health (15.1% vs 9.9%) and limiting long-term conditions (34.5% vs 27.4%) compared to non-LGB + counterparts and were more likely to live in deprived areas (23.2% in the most deprived quintile vs 19.2%). Religious minorities reported better socioeconomic positioning (with 15.8% living in the most deprived quintile vs 20.4% of non-minorities) despite slightly higher rates of poor mental health (12.4% vs 9.9%).

Irish Travellers exhibited substantially worse health and socioeconomic indicators compared to all other population groups. These individuals reported the highest rates of poor mental health (36.1% vs 10.4% in non-Travellers), limiting long-term illness (56.7% vs 28.0%), and poor self-rated health (54.8% reporting fair, bad, or very bad health). Socioeconomically, they experienced the greatest levels of deprivation, with 40.6% living in the most deprived areas and 52.7% living in social housing.

### 3.2 All-cause mortality

Table 1 provides a descriptive overview of all-cause mortality across the USGs. Model-based estimates of all-cause mortality risk are presented in Table 2. Overall, membership of any USG was associated with lower all-cause mortality risk in unadjusted models (HR 0.62, 95% CI 0.61–0.64). The estimate was attenuated after full adjustment but remained below 1.00 (HR 0.91, 95% CI 0.89–0.93).

**Table 2:** Associations between underserved group membership and all-cause mortality in Northern Ireland, with stepwise adjustment for covariates. (Figures represent Hazards ratios & 95% confidence intervals).

Underserved Group (USG)	Unadjusted HR (95 % CI)	+ gender and age band (years)	+ housing tenure and income deprivation	+ general health and LLTI	+ poor mental health
Any USG	**0.62 (0.61**–**0.64)∗∗**	**0.91 (0.89**–**0.93)∗∗**	**0.89 (0.87**–**0.91)∗∗**	**0.91 (0.88**–**0.93)∗∗**	**0.91 (0.89**–**0.93)∗∗**
Non-white	**0.19 (0.16**–**0.22)∗∗**	**0.52 (0.45**–**0.61)∗∗**	**0.50 (0.43**–**0.58)∗∗**	**0.61 (0.54**–**0.70)∗∗**	**0.60 (0.53**–**0.69)∗∗**
Migrants	**0.24 (0.22**–**0.26)∗∗**	**0.68 (0.62**–**0.74)∗∗**	**0.65 (0.60**–**1.27)∗∗**	**0.87 (0.81**–**0.94)∗∗**	**0.84 (0.78**–**0.91)∗∗**
Limited English Proficiency	**0.41 (0.35**–**0.48)∗∗**	**0.72 (0.61**–**0.85)∗∗**	**0.64 (0.54**–**0.76)∗∗**	**0.76 (0.66**–**0.88)∗∗**	**0.72 (0.63**–**0.84)∗∗**
LGB+	**1.24 (1.20**–**1.29)∗∗**	**1.09 (1.05**–**1.13)∗∗**	**1.05 (1.01**–**1.09)∗**	0.99 (0.96 – 1.02)	0.99 (0.96 – 1.02)
Religious minorities	**0.42 (0.40**–**0.44)∗∗**	**0.76 (0.73**–**0.79)∗∗**	**0.77 (0.74**–**0.80)∗∗**	**0.82 (0.79**–**0.86)∗∗**	**0.83 (0.80**–**0.86)∗∗**
Irish Traveller	0.70 (0.46 – 1.06)	1.50 (0.99 – 2.29)	1.18 (0.78 – 1.79)	0.77 (0.53 – 1.12)	0.83 (0.57 – 1.21)

∗P < 0.05.

∗∗P < 0.001.

LLTI: limiting long-term illness.

In unadjusted models, four out of six USGs had lower mortality risk compared with their respective reference groups (Table 2), with hazard ratios (HR) ranging from HR 0.19 (95% CI 0.16-0.22) for ethnic minorities to HR 0.42 (95% CI 0.40-0.44) for religious minorities. Individuals who identified as LGB+ were the only group with an estimate above 1.00 (HR 1.24, 95% CI 1.20-1.29). Estimates for Irish Travellers were imprecise, likely reflecting limited statistical power. Sequential adjustment for socioeconomic factors, general health, and limiting long-term illness attenuated the results. In fully adjusted models including mental health status, estimates remained below 1.00 for ethnic minorities (HR 0.60, 95% CI 0.53-0.69), migrants (HR 0.84, 95% CI 0.78-0.91), those with limited English proficiency (HR 0.72, 95% CI 0.63-0.84), and religious minorities (HR 0.83, 95% CI 0.80-0.86). For LGB + individuals, the estimate was attenuated towards the null (HR 0.99, 95% CI 0.96-1.02).

### 3.3 Avoidable mortality

Patterns for avoidable mortality were broadly similar to those observed for all-cause mortality (Table 3). Membership of any USG was associated with a lower risk of avoidable mortality in unadjusted models (HR 0.66, 95% CI 0.64-0.68), and estimates remained below 1.00 after full adjustment (HR 0.94, 95% CI 0.91-0.97).

**Table 3:** Associations between underserved group membership and avoidable mortality in Northern Ireland, with stepwise adjustment for covariates. (Figures represent Hazards ratios & 95% confidence intervals).

Underserved Group (USG)	Unadjusted HR (95 % CI)	+ gender and age band (years)	+ housing tenure and income deprivation	+ general health and LLTI	+ poor mental health
Any USG	**0.66 (0.64**–**0.68)∗∗**	**0.95 (0.92**–**0.98)∗∗**	**0.93 (0.89**–**0.96)∗∗**	**0.94 (0.91**–**0.97)∗∗**	**0.94 (0.91**–**0.97)∗∗**
Non-white	**0.22 (0.18**–**0.27)∗∗**	**0.57 (0.48**–**0.69)∗∗**	**0.54 (0.45**–**0.65)∗∗**	**0.67 (0.57**–**0.79)∗∗**	**0.66 (0.56**–**0.78)∗∗**
Migrants	**0.27 (0.24**–**0.30)∗∗**	**0.72 (0.65**–**0.80)∗∗**	**0.68 (0.61**–**0.76)∗∗**	**0.91 (0.83**–**0.99)∗**	**0.88 (0.80**–**0.97)∗**
Limited English Proficiency	**0.46 (0.38**–**0.57)∗∗**	**0.78 (0.64**–**0.96)∗**	**0.66 (0.54**–**0.81)∗∗**	**0.80 (0.67**–**0.95)∗**	**0.76 (0.63**–**0.90)∗**
LGB+	**1.25 (1.20**–**1.31)∗∗**	**1.11 (1.06**–**1.17)∗∗**	**1.05 (1.00**–**1.10)∗**	1.00 (0.96 – 1.05)	1.00 (0.96 – 1.05)
Religious minorities	**0.46 (0.44**–**0.48)∗∗**	**0.81 (0.77**–**0.86)∗∗**	**0.82 (0.78**–**0.87)∗∗**	**0.87 (0.83**–**0.92)∗∗**	**0.88 (0.84**–**0.92)∗∗**
Irish Traveller	n/a	n/a	n/a	n/a	n/a

∗P < 0.05.

∗∗P < 0.001.

n/a: insufficient numbers.

LLTI: limiting long-term illness.

In unadjusted models, ethnic minorities, migrants, those with limited English proficiency, and religious minorities had lower risk of avoidable mortality than their respective reference groups (HRs 0.22-0.46), while LGB + individuals had elevated risk (HR 1.25, 95% CI 1.20-1.31). In fully adjusted models including mental health, estimates for ethnic minorities, migrants, those with limited English proficiency, and religious minorities remained below 1.00 (HRs 0.66-0.88), while the estimate for LGB + individuals was attenuated towards the null. Estimates for Irish Travellers could not be presented due to insufficient numbers.

### 3.4 Deaths of despair

For the composite any USG category, estimates for risk of deaths of despair were close to 1.00 in both the unadjusted (HR 1.08, 95% CI 0.96-1.22) and fully adjusted (HR 1.07, 95% CI 0.94-1.21) models (Table 4).

**Table 4:** Associations between underserved group membership and deaths of despair in Northern Ireland, with stepwise adjustment for covariates. (Figures represent Hazards ratios & 95% confidence intervals).

Underserved Group (USG)	Unadjusted HR (95 % CI)	+ gender and age band (years)	+ housing tenure and income deprivation	+ general health and LLTI	+ poor mental health
Any USG	1.08 (0.96 – 1.22)	1.14 (1.01 – 1.29)	1.06 (0.93 – 1.20)	1.07 (0.94 – 1.21)	1.07 (0.94 – 1.21)
Non-white	**0.41 (0.22**–**0.74)∗**	**0.45 (0.25**–**0.82)∗**	**0.39 (0.21**–**0.70)∗**	**0.51 (0.31**–**0.85)∗**	**0.53 (0.32**–**0.88)∗**
Migrants	**0.56 (0.41**–**0.77)∗∗**	**0.62 (0.45**–**0.85)∗**	**0.52 (0.37**–**0.72)∗∗**	**0.73 (0.55**–**0.97)∗**	0.78 (0.58 – 1.03)
Limited English Proficiency	n/a	n/a	n/a	n/a	n/a
LGB+	**1.42 (1.18**–**1.72)∗∗**	**1.55 (1.28**–**1.88)∗∗**	**1.30 (1.07**–**1.57)∗**	**1.22 (1.03**–**1.45)∗**	**1.21 (1.02**–**1.43)∗**
Religious minorities	0.99 (0.84 – 1.18)	1.03 (0.87 – 1.22)	1.04 (0.88 – 1.23)	0.99 (0.85 – 1.16)	0.99 (0.85 – 1.15)
Irish Traveller	n/a	n/a	n/a	n/a	n/a

∗P < 0.05.

∗∗P < 0.001.

n/a: insufficient numbers.

LLTI: limiting long-term illness.

Ethnic minorities had lower risk across all adjustment steps (fully adjusted HR 0.53, 95% CI 0.32-0.88). LGB + individuals had elevated risk in the unadjusted model (HR 1.42, 95% CI 1.18-1.72), and the estimate remained above 1.00 after full adjustment including mental health (HR 1.21, 95% CI 1.02-1.43). Migrants showed lower risk in unadjusted models (HR 0.56, 95% CI 0.41-0.77), but the estimate was attenuated after additional adjustment for mental health (HR 0.78, 95% CI 0.58-1.03). For religious minorities, estimates were close to 1.00 across all models. Insufficient numbers prevented analysis for those with limited English proficiency and Irish Travellers.

### 3.5 Death by suicide

Membership of any USG was associated with a higher suicide mortality risk in unadjusted models (HR 1.31, 95% CI 1.05-1.65), but estimates were attenuated after adjustment and confidence intervals included 1.00 (HR 1.19, 95% CI 0.94-1.50) (Table 5).

**Table 5:** Associations between underserved group membership and death by suicide in Northern Ireland, with stepwise adjustment for covariates. (Figures represent Hazards ratios & 95% confidence intervals).

Underserved Group (USG)	Unadjusted HR (95 % CI)	+ gender and age band (years)	+ housing tenure and income deprivation	+ general health and LLTI	+ poor mental health
Any USG	**1.31 (1.05**–**1.65)∗**	1.25 (0.99 – 1.57)	1.21 (0.96 – 1.53)	1.21 (0.96 – 1.52)	1.19 (0.94 – 1.50)
Non-white	n/a	n/a	n/a	n/a	n/a
Migrants	0.67 (0.37 – 1.20)	0.65 (0.36 – 1.17)	0.57 (0.31 – 1.04)	0.72 (0.43 – 1.18)	0.80 (0.48 – 1.32)
Limited English Proficiency	n/a	n/a	n/a	n/a	n/a
LGB+	1.26 (0.85 – 1.86)	1.31 (0.89 – 1.95)	1.20 (0.81 – 1.79)	1.06 (0.75 – 1.50)	1.02 (0.72 – 1.44)
Religious minorities	**1.41 (1.05**–**1.90)**	1.30 (0.96 – 1.75)	1.32 (0.98 – 1.79)	1.22 (0.94 – 1.58)	1.18 (0.91 – 1.54)
Irish Traveller	n/a	n/a	n/a	n/a	n/a

∗P < 0.05.

∗∗P < 0.001.

n/a: insufficient numbers.

LLTI: limiting long-term illness.

Religious minorities had higher suicide mortality risk in unadjusted models (HR 1.41, 95% CI 1.05-1.90), but this was attenuated in fully adjusted models (HR 1.18, 95% CI 0.91-1.54). For migrants and LGB + individuals, estimates for suicide mortality risk were close to 1.00 or imprecise across models. Insufficient numbers prevented analysis for ethnic minorities, those with limited English proficiency, and Irish Travellers.

## 4 Discussion

Considering all underserved groups together (“any USG vs none”), membership of at least one USG was linked to lower risks of all-cause and avoidable mortality, but not with deaths of despair or death by suicide. This pattern indicates that minority status in general does not uniformly translate into mortality disadvantage, but aggregation can mask subgroup-specific vulnerabilities. The composite category was intended as a broad descriptive contrast, rather than as a substitute for group-specific analyses. Examining individual groups therefore remains essential for identifying where risks are concentrated. These findings should also be interpreted in light of the fact that underserved identities may intersect, and that single-category analyses may not fully capture the ways in which disadvantage is experienced. When individual groups were examined, most USGs in NI showed lower mortality risks than their reference populations, particularly for all-cause and avoidable mortality. These protective effects were attenuated after sequential adjustment for sociodemographic and health-related factors, suggesting that these characteristics are important to the observed patterning of mortality risk.

The unadjusted models provide valuable insight into mortality risk differences observed in USGs in NI. LGB + individuals had higher risk of all-cause mortality, avoidable mortality, and deaths of despair than their non-LGB + counterparts, while religious minorities had higher suicide mortality risk in unadjusted models. In contrast, ethnic minorities, migrants, and those with limited English proficiency had lower mortality risk across several outcomes compared with their respective reference groups. For Irish Travellers, the small number of observed deaths meant that all-cause mortality estimates should be interpreted cautiously.

For LGB + individuals, our findings align with international evidence documenting elevated mortality risks (Zachry et al., 2025). This is consistent with other quantitative reviews showing higher suicide risk and self-harm among sexual and gender minority populations (Marchi et al., 2022). The association between LGB + status and all-cause mortality risk was attenuated after adjustment for socioeconomic and health-related factors, including mental health. Our descriptive data similarly showed poorer mental health and higher levels of limiting long-term illness in this population. These patterns align with previous work suggesting that social and health-related disadvantage may contribute to mortality inequalities among sexual minority populations (Harkins & Hoffman, 2024; Zeeman et al., 2020). Nevertheless, the estimate for deaths of despair remained elevated after full adjustment, suggesting a distinct pattern that may warrant further attention. However, this composite outcome should be interpreted cautiously. “Deaths of despair” is a contested term, and the causes grouped within it are heterogeneous (Darke et al., 2025). In this study, the category is used pragmatically as a composite grouping of suicide, alcohol-related deaths, and drug-related deaths, rather than as implying a single underlying mechanism. Notably, while the composite deaths of despair estimate remained elevated for LGB + individuals after full adjustment, the corresponding estimate for suicide alone was close to the null (HR 1.02, 95% CI 0.72-1.44), suggesting that the excess risk within the composite may be driven by alcohol-related or drug-related causes rather than by suicide. However, as the relative contribution of each constituent cause cannot be separately reported due to statistical disclosure control requirements, this interpretation should be treated with caution. Minority stress theory may help explain this residual risk, as chronic exposure to stigma, prejudice, and discrimination may contribute to substance use and self-destructive behaviours through pathways not fully captured by our mental health measures (Bränström & Pachankis, 2018; Frost et al., 2015; Hatzenbuehler et al., 2024; Meyer, 2003; Puckett et al., 2015).

Among religious minorities, we observed consistently lower all-cause and avoidable mortality risks, despite a notably elevated suicide risk in unadjusted models. This complex pattern aligns with research on religious affiliation's protective effects through social support, community integration, and health-promoting behaviours (Koenig et al., 2023; Poorolajal et al., 2021; VanderWeele, 2017). However, the heterogeneity within our “religious minority” category likely underpins the contrasting patterns. Our findings differ notably from O'Reilly and Rosato's NI study, which examined suicide risk across different religious groups between 2001 and 2009 (O'Reilly & Rosato, 2015). O'Reilly and Rosato found similar suicide risk across Catholics, Protestants, and the non-religious after adjusting for socioeconomic factors, but noted lower risk among conservative Christians. They also observed age-specific patterns - higher risk among young Catholics and lower risk in older Catholics - highlighting more nuanced relationships than our broader categorisation captured. Differences in findings likely reflect methodological contrasts, including O'Reilly's more detailed religious categories and our analysis of a more recent, increasingly secular period (2021–2023).

Lower mortality risk was observed among both ethnic minority and migrant groups, but these patterns should not be assumed to reflect the same underlying processes. The lower mortality risk observed among migrants is consistent with the well-documented “healthy migrant effect”, whereby new arrivals may initially exhibit more favourable health profiles than host populations (Bhopal et al., 2018; Shor & Roelfs, 2021; Wallace & Darlington-Pollock, 2022; Wallace & Wilson, 2022). This pattern persisted after adjustment, suggesting that it is not fully accounted for by the covariates included in the present analysis. The composition of NI's migrant population likely contributes to these findings, as the region hosts relatively small numbers of asylum seekers compared with its wider migrant population. Law Centre (2024); Northern Ireland Assembly Research and Information Service (RaISe), 2025 Asylum seekers and refugees typically face greater barriers to healthcare and heightened mental health needs, which may help explain differing mortality patterns across groups. (Asif & Kienzler, 2022; Dumke et al., 2024; Law Centre, 2024; Mental Health Foundation, 2025; Northern Ireland Assembly Research and Information Service (RaISe), 2025) Additionally, NI's relatively recent immigration patterns may mean we are observing migrants in the early stages of settlement, when the healthy migrant effect is strongest (Rechel et al., 2013). However, the present analysis does not distinguish between first- and later-generation ethnic minority populations, and this is important for interpreting the lower mortality risk observed among ethnic minority groups. The apparent advantage in the ethnic minority category may partly reflect the inclusion of first-generation migrants, and therefore should be interpreted cautiously. The relatively small size of ethnic minority communities might foster what Portes and Zhou term "segmented assimilation," wherein certain immigrant groups maintain protective cultural practices while selectively adapting to the host society (Portes & Zhou, 1993).

The intersection between migrant status and language proficiency represents another important dimension in understanding these mortality patterns. Those with limited English proficiency had lower risk of deaths of despair after full adjustment, despite potential barriers to healthcare access. This finding aligns with Alarcón et al.'s "linguistic isolation hypothesis” (Alarcón et al., 2016), which suggests that limited host-language proficiency may temporarily shield migrants from acculturative stress by maintaining stronger connections with traditional support networks and cultural practices.

For Irish Travellers, the small number of deaths limited precision in the all-cause mortality analysis, making it difficult to draw firm conclusions about mortality risk. However, our baseline descriptive data indicated poorer mental health and a greater burden of long-term illness in this population, consistent with longstanding evidence of health disadvantage (All Ireland Traveller Health Study Team, 2010; Dagli & Webb, 2025; Kennedy et al., 2023; Knipe et al., 2024; McKey et al., 2022).

### 4.1 Strengths and limitations

Our analysis benefits from several strengths. The population-wide coverage and linkage to high-quality mortality data allowed us to examine multiple outcomes while minimising selection bias. The inclusion of small-area deprivation data also allowed the analysis to account for the wider socioeconomic context in which individuals were living. The Census provided standardised information on characteristics such as ethnicity, religion, and sexual orientation - data rarely collected in other administrative datasets. Furthermore, the legal requirement to participate in the Census contributed to comprehensive population coverage, enhancing the representativeness of our findings. By including both individual USGs and a composite “any USG” category, we were able to consider whether mortality risks are shaped by minority status in general or by specific vulnerabilities. While the aggregated analysis suggested broadly protective patterns for mortality, the group-specific analyses highlighted that risks are unevenly distributed, with certain populations (e.g. LGB + individuals) facing distinct disadvantages. This dual approach provides a fuller picture but also underscores the limitations of treating USGs as a single analytical category.

Nevertheless, several important limitations warrant consideration. Our binary categorisation of USGs, though methodologically necessary, likely masks significant within-group heterogeneity in social circumstances and health outcomes. In addition, the groups included in this study were limited to those identifiable within Census data, and therefore do not capture all populations who may experience substantial disadvantage. Several covariates included in later models, particularly socioeconomic and health-related factors, may lie on the pathway between USG membership and mortality risk. These models should therefore be interpreted as showing how associations change across adjustment stages rather than as estimating direct effects. Administrative data, while comprehensive, cannot capture the nuanced lived experiences of discrimination or healthcare access barriers that may influence mortality risk. Avoidable mortality is a constructed category and its definition remains open to debate. Although the OECD/Eurostat classification provides a widely used and transparent framework, it may not capture all deaths that are socially preventable, particularly those linked to broader structural disadvantage. The accuracy of self-reported variables may be influenced by Census collection methods, particularly as forms may be completed by a single household reference person on behalf of all household members. This proxy reporting may affect the reliability of certain measures, especially for sensitive characteristics such as LGB + status, where individuals may not have disclosed their identity to household members, or where social desirability bias may influence reporting. Additionally, these USGs often overlap, and the present analysis does not capture how multiple forms of disadvantage may intersect within individuals. This is important because mortality risk may be shaped not only by membership of a single group, but by the combined and interacting effects of multiple marginalised identities. Further work using multilevel approaches is underway to examine intersectional patterning more directly. Our follow-up period of 33 months may be insufficient to fully capture longer-term mortality patterns, particularly for smaller population subgroups like Irish Travellers. Furthermore, information on emigration was not available, which may have resulted in some unobserved loss to follow-up. However, given the relatively short follow-up period, the impact on the estimates is likely to be limited.

## 5 Conclusions

The contrasting mortality patterns across USGs indicate that a single approach to addressing inequality is unlikely to reflect the different risk profiles observed across groups. While associations were attenuated after accounting for sociodemographic and health factors, the estimate for deaths of despair among LGB + individuals remained elevated, suggesting that this group may warrant particular attention in mental health and substance use policy and practice. The aggregated “any USG” analyses showed broadly protective associations for all-cause and avoidable mortality, but this masks the distinct vulnerabilities faced by particular groups. Addressing inequalities is likely to require both broad strategies to tackle structural disadvantage and tailored approaches for especially at-risk populations.

This study illustrates the value of Census-linked administrative data in examining mortality among USGs, but also highlights the urgent need for improved collection of protected characteristics in routine health datasets (Bozorgmehr et al., 2023; ONS, 2024; Razieh et al., 2025). Enhanced data infrastructure could provide more timely evidence on evolving health needs without depending solely on decennial Census data, enabling more responsive and equitable health services. Future research should explore intersectionality between USGs, examine potential mechanisms underlying these mortality patterns, assess whether these associations persist over longer follow-up periods, and build on the patterns identified here by examining alcohol-related and drug-related mortality separately where disclosure requirements permit. Complementary qualitative research could provide valuable insights into lived experiences and inform culturally appropriate interventions that address the unique health challenges faced by Northern Ireland's increasingly diverse population.

## CRediT authorship contribution statement

**Emma Ross:** Writing – review & editing, Writing – original draft, Visualization, Methodology, Formal analysis, Data curation. **Sarah McKenna:** Writing – review & editing, Methodology, Data curation. **Aideen Maguire:** Writing – review & editing, Supervision, Methodology, Funding acquisition, Conceptualization.

## Ethics approval statement

The NIMS database has ethical approval from the Office for Research Ethics Committees Northern Ireland (ORECNI) (REC Reference Number 22/NI/0042). This study was approved by the Northern Ireland Longitudinal Study Research Approvals Group (RAG). All data were de-identified and handled in accordance with the Data Protection Act 2018 and the Northern Ireland Statistics and Research Agency's (NISRA's) data security protocols.

## Ethical Statement

Ethical approval and data governance.

The Northern Ireland Mortality Study (NIMS) database has ethical approval from the Office for Research Ethics Committees Northern Ireland (ORECNI) (REC Reference Number: 22/NI/0042). This study was approved by the Northern Ireland Longitudinal Study Research Approvals Group (RAG).

All data were fully de-identified prior to analysis and were handled in accordance with the Data Protection Act 2018 and the Northern Ireland Statistics and Research Agency's (NISRA's) data security and disclosure control protocols. Analyses were conducted within a secure research environment, and no direct contact with study participants occurred.

Informed consent was not required, as the study used de-identified administrative data collected for statutory purposes.

## Funding

This work was supported by the UKRI's Administrative Data Research Centre Northern Ireland (ESW010240/1), Medical Research Council Fellowship (MR/P014631/1/) and Health Data Research UK/DATAMIND (MR/W014386/1). For the purpose of open access, the author has applied a Creative Commons Attribution (CC BY) licence to any Author Accepted Manuscript version arising from this submission.

## Declaration of competing interest

The authors declare that they have no known competing financial interests or personal relationships that could have appeared to influence the work reported in this paper.

## Data Availability

The authors do not have permission to share data.
