# Supplementary material for: Enhancing population-wide administrative data to better understand mortality risk in underserved groups
Source: SSM Popul Health. 2026 May 14;34:101933. doi: 10.1016/j.ssmph.2026.101933 (PMC13199807; doi:10.1016/j.ssmph.2026.101933)
Supplement: Multimedia component 1 [file mmc1.docx]

Supplementary Materials

**Supplementary Table 1. ICD-10 codes identifying avoidable causes of mortality**

| **Group** | **Cause of death** | **ICD-10 Code** |
| --- | --- | --- |
|  | | |
| Infectious diseases | Intestinal diseases | A00-A09 |
|  | Diphtheria, Tetanus, Poliomyelitis | A35, A36, A80 |
|  | Whooping cough | A37 |
|  | Meningococcal infection | A39 |
|  | Sepsis due to streptococcus pneumonia and sepsis due to hemophilus influenzae | A40.3, A41.3 |
|  | Haemophilus influenza infections | A49.2 |
|  | Sexually transmitted infections (except HIV/AIDS) | A50-A60, A63, A64 |
|  | Varicella | B01 |
|  | Measles | B05 |
|  | Rubella | B06 |
|  | Viral Hepatitis | B15-B19 |
|  | HIV/AIDS | B20-B24 |
|  | Malaria | B50-B54 |
|  | Haemophilus and pneumococcal meningitis | G00.0, G00.1 |
|  | Tuberculosis | A15-A19, B90, J65 |
|  | | |
| Cancer | Lip, oral cavity and pharynx cancer | C00-C14 |
|  | Oesophageal cancer | C15 |
|  | Stomach cancer | C16 |
|  | Liver cancer | C22 |
|  | Lung cancer | C33-C34 |
|  | Mesothelioma | C45 |
|  | Skin (melanoma) cancer | C43 |
|  | Bladder cancer | C67 |
|  | Cervical cancer | C53 |
|  | Colorectal cancer | C18-C21 |
|  | Breast cancer (female only) | C50 |
|  | Uterus cancer | C54,C55 |
|  | Testicular cancer | C62 |
|  | Thyroid cancer | C73 |
|  | Hodgkin's disease | C81 |
|  | Lymphoid leukaemia | C91.0, C91.1 |
|  | Benign neoplasm | D10-D36 |
|  | | |
| Endocrine and metabolic diseases | Nutritional deficiency anaemia | D50-D53 |
|  | Diabetes mellitus | E10-E14 |
|  | Thyroid disorders | E00-E07 |
|  | Adrenal disorders | E24-E25 (except E24.4), E27 |
|  | | |
| Diseases of the nervous system | Epilepsy | G40,G41 |
|  | | |
| Diseases of the circulatory system | Aortic aneurysm | I71 |
|  | Hypertensive diseases | I10-I13, I15 |
|  | Ischaemic heart diseases | I20-I25 |
|  | Cerebrovascular diseases | I60-I69 |
|  | Other atherosclerosis | I70, I73.9 |
|  | Rheumatic and other heart disease | I00-I09 |
|  | Venous thromboembolism | I26, I80, I82.9 |
|  | | |
| Diseases of the respiratory system | Influenza | J09-J11 |
|  | Pneumonia due to Streptococcus pneumonia or Haemophilus influenza | J13-J14 |
|  | Chronic lower respiratory diseases | J40-J44 |
|  | Lung diseases due to external agents | J60-J64, J66-J70, J82, J92 |
|  | Upper respiratory infections | J00-J06, J30-J39 |
|  | Pneumonia, not elsewhere classified or organism unspecified | J12, J15, J16-J18 |
|  | Acute lower respiratory infections | J20-J22 |
|  | Asthma and bronchiectasis | J45-J47 |
|  | Adult respiratory distress syndrome | J80 |
|  | Pulmonary oedema | J81 |
|  | Abscess of lung and mediastinum pyothorax | J85, J86 |
|  | Other pleural disorders | J90, J93, J94 |
|  | | |
| Diseases of the digestive system | Gastric and duodenal ulcer | K25-K28 |
|  | Appendicitis | K35-K38 |
|  | Abdominal hernia | K40-K46 |
|  | Cholelithiasis and cholecystitis | K80-K81 |
|  | Other diseases of gallbladder or biliary tract | K82-K83 |
|  | Acute pancreatitis | K85.0,1,3,8,9 |
|  | Other diseases of pancreas | K86.1,2,3,8,9 |
|  | | |
| Diseases of the genitourinary system | Nephritis and nephrosis | N00-N07 |
|  | Obstructive uropathy | N13,N20-N21, N35 |
|  | Renal failure | N17-N19 |
|  | Renal colic | N23 |
|  | Disorders resulting from renal tubular dysfunction | N25 |
|  | Unspecified contracted kidney, small kidney of unknown cause | N26-N27 |
|  | Inflammatory diseases of genitourinary system | N34.1,N70-N73,N75.0,N75.1,N76.4,6 |
|  | Prostatic hyperplasia | N40 |
|  | | |
| Pregnancy, childbirth and perinatal period | Tetanus neonatorum | A33 |
|  | Obstetrical tetanus | A34 |
|  | Pregnancy, childbirth and the puerperium | O00-O99 |
|  | Certain conditions originating in the perinatal period | P00-P96 |
|  | | |
| Congenital malformations | Certain congenital malformations (neural tube defects) | Q00, Q01, Q05 |
|  | Congenital malformations of the circulatory system (heart defects) | Q20-Q28 |
|  | | |
| Adverse effects of medical and surgical care | Drugs, medicaments and biological substances causing adverse effects in therapeutic use | Y40-Y59 |
|  | Misadventures to patients during surgical and medical care | Y60-Y69,Y83-Y84 |
|  | Medical devices associated with adverse incidents in diagnostic and therapeutic use | Y70–Y82 |
|  | | |
| Injuries | Transport Accidents | V01-V99 |
|  | Accidental Injuries | W00-X39, X46-X59 |
|  | Intentional self-harm | X66-X84 |
|  | Event of undetermined intent | Y16-Y34 |
|  | Assault | X86-Y09 |
|  | | |
| Alcohol-related and drug-related deaths | Alcohol-specific disorders and poisonings | E24.4, F10, G31.2, G62.1, G72.1, I42.6, K29.2, K70, K85.2, K86.0, Q86.0, R78.0, X45, X65, Y15 |
|  | Other alcohol-related disorders | K73, K74.0-K74.2, K74.6 |
|  | Drug disorders and poisonings | F11-F16, F18-F19, X40-X44, X85, Y10-Y14 |
|  | Intentional self-poisoning by drugs | X60-X64 |
|  | | |
| Provisional assignment of new diseases | COVID-19 | U07.1- U07.2 |

**Supplementary Figure 1. Directed acyclic graph (DAG) illustrating the simplified analytical framework underpinning the sequential models of associations between underserved group membership and mortality outcomes.**


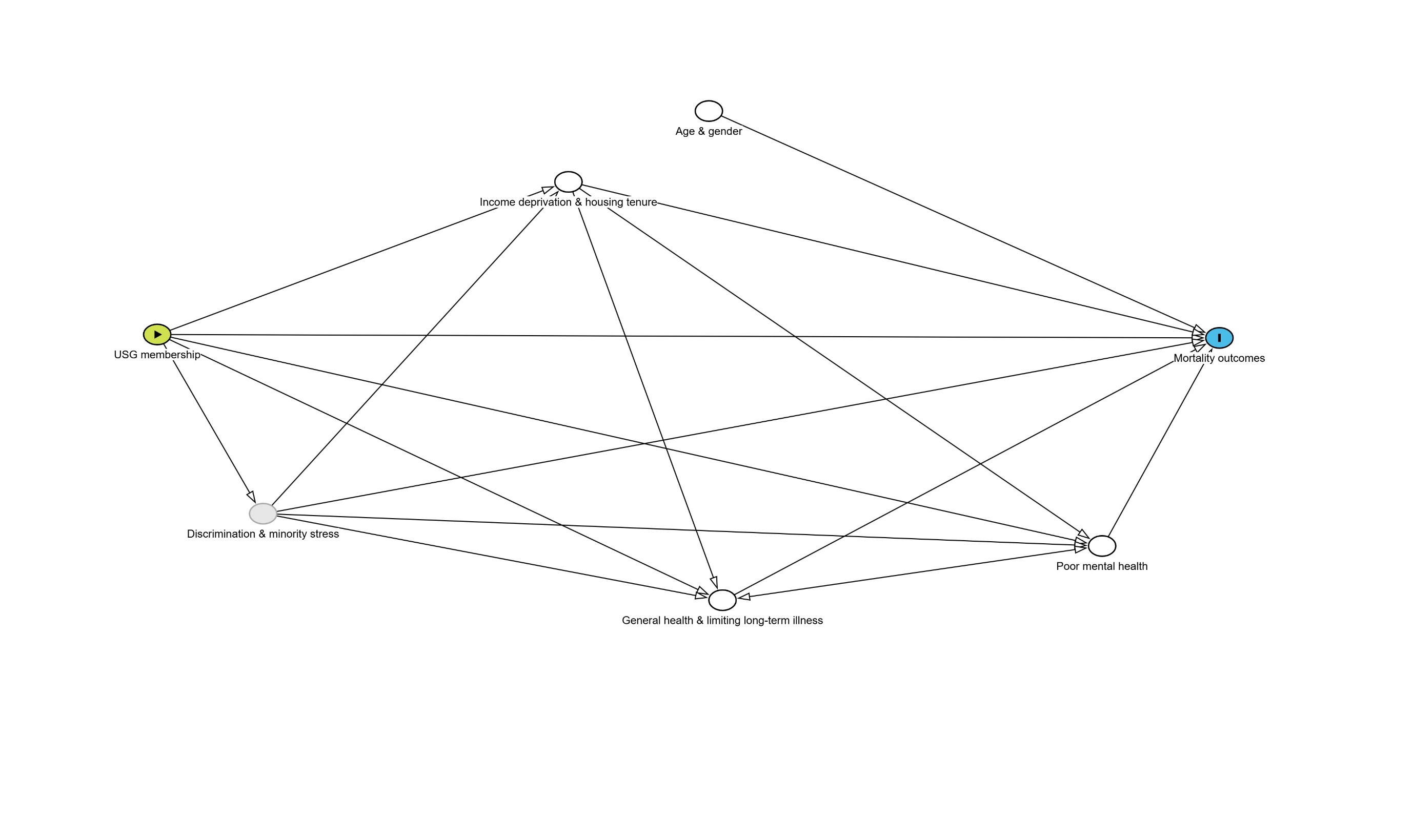


**Notes:** This DAG is included to clarify the assumed relationships underlying the sequential adjustment strategy. Age and gender were treated as baseline covariates. Income deprivation/housing tenure, general health/limiting long-term illness, and poor mental health were treated as pathway variables that may contribute to the association between underserved group membership and mortality outcomes. Discrimination and minority stress are shown as unmeasured influences. The diagram is intentionally simplified for clarity. The relationship between poor mental health and general health/limiting long-term illness is represented as bidirectional, reflecting robust evidence that each influences the other. This means the diagram is not a strict directed acyclic graph, but is included to illustrate the assumed structure of the analytical framework and does not imply that the present study estimates causal effects.

**Supplementary Table 2. Sociodemographic and Health Characteristics by Ethnic Minority Status in Northern Ireland**

| **Characteristic** | | **White (n=1,422,336)** | **Non-White (n=41,123)** | **p-value** |
| --- | --- | --- | --- | --- |
|  | | | | |
| **Gender** | Male | 689,093 (48.5) | 20,714 (50.4) | <0.001 |
|  | Female | 733, 243 (51.6) | 20,409 (49.6) |  |
|  | | | | |
| **Age band (years)** | Under 25 | 181,950 (12.8) | 7,287 (17.7) | <0.001 |
|  | 25-44 | 455,647 (32.0) | 20,894 (50.8) |  |
|  | 45-64 | 476,006 (33.5) | 10,744 (26.1) |  |
|  | 65+ | 308,733 (21.7) | 2,198 (5.3) |  |
|  | | | | |
| **Housing tenure** | Owned outright | 1,047,136 (73.6) | 18,723 (45.5) | <0.001 |
|  | Privately rented | 201, 577 (14.2) | 16,403 (39.9) |  |
|  | Socially rented | 173, 623 (12.2) | 5,997 (14.6) |  |
|  | | | | |
| **Income deprivation quintile** | Least deprived | 269,842 (19.0) | 9,226 (22.4) | <0.001 |
|  | 2 | 288, 854 (20.3) | 6,314 (15.4) |  |
|  | 3 | 289, 897 (20.4) | 8,033 (19.5) |  |
|  | 4 | 296,172 (20.8) | 8,533 (20.8) |  |
|  | Most deprived | 277, 571 (19.5) | 9,017 (21.9) |  |
|  | | | | |
| **General health** | Very good | 589,187 (41.4) | 20,444 (49.7) | <0.001 |
|  | Good | 466, 255 (32.8) | 14,362 (34.9) |  |
|  | Fair | 233, 762 (16.4) | 4,325 (10.5) |  |
|  | Bad | 99, 730 (7.0) | 1,476 (3.6) |  |
|  | Very bad | 33,402 (2.4) | 516 (1.30) |  |
|  | | | | |
| **Limiting long-term illness** | No | 1,018,812 (71.6) | 34, 941 (85.0) | <0.001 |
|  | Yes – activities limited a little | 213, 384 (15.0) | 3,798 (9.2) |  |
|  | Yes – activities limited a lot | 190, 140 (13.4) | 2,384 (5.8) |  |
|  | | | | |
| **Poor mental health** | No | 1,273,019 (89.5) | 38, 607 (93.9) | <0.001 |
|  | Yes | 149, 317 (10.5) | 2,516 (6.1) |  |
|  | | | | |

**Supplementary Table 3. Sociodemographic and Health Characteristics by Migrant Status in Northern Ireland**

| **Characteristic** | | **Non-migrant (n=1,359,417)** | **Migrant (n=104,042)** | **p-value** |
| --- | --- | --- | --- | --- |
|  | | | | |
| **Gender** | Male | 659,678 (48.5) | 50,129 (48.2) | 0.032 |
|  | Female | 699,739 (51.5) | 53, 913 (51.8) |  |
|  | | | | |
| **Age band (years)** | Under 25 | 175,601 (12.9) | 13,636 (13.1) | <0.001 |
|  | 25-44 | 420,143 (30.9) | 56,398 (54.2) |  |
|  | 45-64 | 458,076 (33.7) | 28,674 (27.6) |  |
|  | 65+ | 305, 597 (22.5) | 5,334 (5.1) |  |
|  | | | | |
| **Housing tenure** | Owned outright | 1,022,509 (75.2) | 43,350 (41.7) | <0.001 |
|  | Privately rented | 168,655 (12.4) | 49,325 (47.4) |  |
|  | Socially rented | 168,253 (12.4) | 11,367 (10.9) |  |
|  | | | | |
| **Income deprivation quintile** | Least deprived | 259,436 (19.1) | 19,632 (18.9) | <0.001 |
|  | 2 | 279,285 (20.5) | 15,883 (15.3) |  |
|  | 3 | 277,106 (20.4) | 20,824 (20.0) |  |
|  | 4 | 280,737 (20.7) | 23,968 (23.0) |  |
|  | Most deprived | 262,853 (19.3) | 23,735 (22.8) |  |
|  | | | | |
| **General health** | Very good | 559,935 (41.2) | 49,696 (47.8) | <0.001 |
|  | Good | 440,666 (32.4) | 39,951 (38.4) |  |
|  | Fair | 228,057 (16.8) | 10,030 (9.6) |  |
|  | Bad | 97,723 (7.2) | 3,483 (3.4) |  |
|  | Very bad | 33,036 (2.4) | 882 (0.9) |  |
|  | | | | |
| **Limiting long-term illness** | No | 963,686 (70.9) | 90,067 (86.6) | <0.001 |
|  | Yes – activities limited a little | 208,160 (15.3) | 9,022 (8.7) |  |
|  | Yes – activities limited a lot | 187,571 (13.8) | 4,953 (4.8) |  |
|  | | | | |
| **Poor mental health** | No | 1,212,860 (89.2) | 98,766 (94.9) | <0.001 |
|  | Yes | 146,557 (10.8) | 5,276 (5.1) |  |
|  | | | | |

**Supplementary Table 4. Sociodemographic and Health Characteristics by English Language Proficiency Status in Northern Ireland**

| **Characteristic** | | **High English Proficiency (n=1,446,278)** | **Low English Proficiency (n=17,181)** | **p-value** |
| --- | --- | --- | --- | --- |
|  | | | | |
| **Gender** | Male | 701,718 (48.5) | 9,092 (52.9) | <0001 |
|  | Female | 744,560 (51.5) | 8,089 (47.1) |  |
|  | | | | |
| **Age band (years)** | Under 25 | 188,141 (13.0) | 1,096 (6.4) | <0.001 |
|  | 25-44 | 469,283 (32.5) | 7,258 (42.2) |  |
|  | 45-64 | 479,474 (33.2) | 7,276 (42.4) |  |
|  | 65+ | 309,380 (21.4) | 1,551 (9.0) |  |
|  | | | | |
| **Housing tenure** | Owned outright | 1,061,561 (73.4) | 4,298 (25.0) | <0.001 |
|  | Privately rented | 208,036 (14.4) | 9,944 (57.9) |  |
|  | Socially rented | 176,681 (12.2) | 2,939 (17.1) |  |
|  | | | | |
| **Income deprivation quintile** | Least deprived | 276,716 (19.0) | 2,352 (13.7) | <0.001 |
|  | 2 | 293,262 (20.3) | 1,906 (11.1) |  |
|  | 3 | 294,636 (20.4) | 3,294 (19.2) |  |
|  | 4 | 299,941 (20.7) | 4,764 (27.7) |  |
|  | Most deprived | 281,723 (19.5) | 4,865 (28.3) |  |
|  | | | | |
| **General health** | Very good | 604,736 (41.8) | 4,895 (28.5) | <0.001 |
|  | Good | 473, 001 (32.7) | 7,616 (44.3) |  |
|  | Fair | 235,078 (16.3) | 3,009 (17.5) |  |
|  | Bad | 99,878 (6.9) | 1,328 (7.7) |  |
|  | Very bad | 33,585 (2.3) | 333 (1.9) |  |
|  | | | | |
| **Limiting long-term illness** | No | 1,040,406 (71.9) | 13,347 (77.7) | <0.001 |
|  | Yes – activities limited a little | 214,891 (14.9) | 2,291 (13.3) |  |
|  | Yes – activities limited a lot | 190,981 (13.2) | 1,543 (9.0) |  |
|  | | | | |
| **Poor mental health** | No | 1,295,216 (89.6) | 16,410 (95.5) | <0.001 |
|  | Yes | 151,062 (10.4) | 771 (4.5) |  |
|  | | | | |

**Supplementary Table 5. Sociodemographic and Health Characteristics by LBG+ Status in Northern Ireland**

| **Characteristic** | | **Not LGB+ (n=1,327,030)** | **LGB+ (n=136,429)** | **p-value** |
| --- | --- | --- | --- | --- |
|  | | | | |
| **Gender** | Male | 644,570 (48.6) | 65,237 (47.8) | <0.001 |
|  | Female | 682,460 (51.4) | 71,192 (52.2) |  |
|  | | | | |
| **Age band (years)** | Under 25 | 166,343 (12.5) | 22,894 (16.8) | <0.001 |
|  | 25-44 | 432,051 (32.6) | 44,490 (32.6) |  |
|  | 45-64 | 452,511 (34.1) | 34,239 (25.1) |  |
|  | 65+ | 276,125 (20.8) | 34,806 (25.5) |  |
|  | | | | |
| **Housing tenure** | Owned outright | 983,450 (74.1) | 82,409 (60.4) | <0.001 |
|  | Privately rented | 187,370 (14.1) | 30,610 (22.4) |  |
|  | Socially rented | 156,210 (11.8) | 23,410 (17.2) |  |
|  | | | | |
| **Income deprivation quintile** | Least deprived | 256,105 (19.3) | 22,963 (16.8) | <0.001 |
|  | 2 | 270, 605 (20.4) | 24,563 (18.0) |  |
|  | 3 | 271,082 (20.4) | 26,848 (19.7) |  |
|  | 4 | 274,360 (20.7) | 30,345 (22.2) |  |
|  | Most deprived | 254,878 (19.2) | 31,710 (23.2) |  |
|  | | | | |
| **General health** | Very good | 562,493 (42.4) | 47,138 (34.6) | <0.001 |
|  | Good | 435,055 (32.8) | 45,562 (33.4) |  |
|  | Fair | 210,893 (15.9) | 27,194 (19.9) |  |
|  | Bad | 89,210 (6.7) | 11,996 (8.8) |  |
|  | Very bad | 29,379 (2.2) | 4,539 (3.3) |  |
|  | | | | |
| **Limiting long-term illness** | No | 964,387 (72.7) | 89,366 (65.5) | <0.001 |
|  | Yes – activities limited a little | 193,254 (14.6) | 23,928 (17.5) |  |
|  | Yes – activities limited a lot | 169,389 (12.8) | 23,135 (17.0) |  |
|  | | | | |
| **Poor mental health** | No | 1,195,802 (90.1) | 115,824 (84.9) | <0.001 |
|  | Yes | 131,228 (9.9) | 20,605 (15.1) |  |
|  | | | | |

**Supplementary Table 6. Sociodemographic and Health Characteristics by Religious Minority Status in Northern Ireland**

| **Characteristic** | | **Does not belong to minority religion (n=1,201,129)** | **Belongs to minority religion (n=262,330)** | **p-value** |
| --- | --- | --- | --- | --- |
|  | | | | |
| **Gender** | Male | 567,215 (47.2) | 142,592 (54.4) | <0.001 |
|  | Female | 633,914 (52.8) | 119,738 (45.6) |  |
|  | | | | |
| **Age band (years)** | Under 25 | 146,981 (12.2) | 42,256 (16.1) | <0.001 |
|  | 25-44 | 361,592 (30.1) | 114,949 (43.8) |  |
|  | 45-64 | 409,800 (34.1) | 76,950 (29.3) |  |
|  | 65+ | 282,756 (23.5) | 28,175 (10.7) |  |
|  | | | | |
| **Housing tenure** | Owned outright | 897,429 (74.7) | 168,430 (64.2) | <0.001 |
|  | Privately rented | 155,327 (12.9) | 62,653 (23.9) |  |
|  | Socially rented | 148,373 (12.4) | 31,247 (11.9) |  |
|  | | | | |
| **Income deprivation quintile** | Least deprived | 214,801 (17.9) | 64,267 (24.5) | <0.001 |
|  | 2 | 235,973 (19.7) | 59,195 (22.6) |  |
|  | 3 | 246,587 (20.5) | 51,343 (19.6) |  |
|  | 4 | 258,723 (21.5) | 45,982 (17.5) |  |
|  | Most deprived | 245,045 (20.4) | 41,543 (15.8) |  |
|  | | | | |
| **General health** | Very good | 498,048 (41.5) | 111,583 (42.5) | <0.001 |
|  | Good | 387,363 (32.3) | 93,254 (35.6) |  |
|  | Fair | 200,135 (16.7) | 37,952 (14.5) |  |
|  | Bad | 86,352 (7.2) | 14,854 (5.7) |  |
|  | Very bad | 29,231 (2.4) | 4,687 (1.8) |  |
|  | | | | |
| **Limiting long-term illness** | No | 850,618 (70.8) | 203,135 (77.4) | <0.001 |
|  | Yes – activities limited a little | 183,033 (15.2) | 34,149 (13.0) |  |
|  | Yes – activities limited a lot | 167,478 (13.9) | 25,046 (9.6) |  |
|  | | | | |
| **Poor mental health** | No | 1,081,739 (90.1) | 229,887 (87.6) | <0.001 |
|  | Yes | 119,390 (9.9) | 151,833 (12.4) |  |
|  | | | | |

**Supplementary Table 7. Sociodemographic and Health Characteristics of Irish Traveller and Non-Traveller Populations in Northern Ireland**

| **Characteristic** | | **Not Irish Traveller (n=1,461,906)** | **Irish Traveller (n=1,553)** | **p-value** |
| --- | --- | --- | --- | --- |
|  | | | | |
| **Gender** | Male | 709,067 (48.5) | 740 (47.7) | 0.501 |
|  | Female | 752,839 (51.5) | 813 (52.4) |  |
|  | | | | |
| **Age band (years)** | Under 25 | 188,842 (12.9) | 395 (25.4) | <0.001 |
|  | 25-44 | 475,891 (32.6) | 650 (41.9) |  |
|  | 45-64 | 486,369 (33.3) | 381 (24.5) |  |
|  | 65+ | 310,804 (21.3) | 127 (8.2) |  |
|  | | | | |
| **Housing tenure** | Owned outright | 1,065,449 (72.9) | 410 (26.4) | <0.001 |
|  | Privately rented | 217,656 (14.9) | 324 (20.9) |  |
|  | Socially rented | 178,801 (12.2) | 819 (52.7) |  |
|  | | | | |
| **Income deprivation quintile** | Least deprived | 278,989 (19.1) | 79 (5.1) | <0.001 |
|  | 2 | 295,045 (20.2) | 123 (7.9) |  |
|  | 3 | 297,586 (20.4) | 344 (22.2) |  |
|  | 4 | 304,328 (20.8) | 377 (24.3) |  |
|  | Most deprived | 285,958 (19.6) | 630 (40.6) |  |
|  | | | | |
| **General health** | Very good | 609,312 (41.7) | 319 (20.5) | <0.001 |
|  | Good | 480,235 (32.9) | 382 (24.6) |  |
|  | Fair | 237,700 (16.3) | 387 (24.9) |  |
|  | Bad | 100,895 (6.9) | 311 (20.0) |  |
|  | Very bad | 33,764 (2.3) | 154 (9.9) |  |
|  | | | | |
| **Limiting long-term illness** | No | 1,053,080 (72.0) | 673 (43.3) | <0.001 |
|  | Yes – activities limited a little | 216,837 (14.8) | 345 (22.2) |  |
|  | Yes – activities limited a lot | 191,989 (13.1) | 535 (34.5) |  |
|  | | | | |
| **Poor mental health** | No | 1,310,633 (89.7) | 993 (63.9) | <0.001 |
|  | Yes | 151,273 (10.4) | 560 (36.1) |  |
|  | | | | |
